# In vitro MSC function is related to clinical reaction in vivo

**DOI:** 10.1186/s13287-018-1037-4

**Published:** 2018-11-08

**Authors:** Aileen L. Rowland, Jiajie Jessica Xu, Amanda Jo Joswig, Carl A. Gregory, Douglas F. Antczak, Kevin J. Cummings, Ashlee E. Watts

**Affiliations:** 1 Department of Large Animal Clinical Sciences, Texas A&M University, College Station, TX USA; 2 Department of Molecular and Cellular Medicine, Institute for Regenerative Medicine, Texas A&M Health Science Center, College Station, TX USA; 3 Department of Microbiology and Immunology, Cornell University, Ithaca, NY USA; 4 Department of Population Medicine and Diagnostic Sciences, Cornell University, Ithaca, NY USA

**Keywords:** MSC, Bone marrow, Horse, Joint, Intra-articular, Allogeneic, Immunogenicity, Trilineage differentiation, Stemness

## Abstract

**Background:**

We recently demonstrated that intracellular xenogen-contaminated autologous MSCs (FBS) and non-xenogen-contaminated allogeneic (ALLO) MSCs caused an adverse clinical response after repeated intra-articular injection in horses, whereas autologous (AUTO) MSCs did not. Our current objective was to use clinical data from the previous study to compare MSC stemness against adverse response indicated by synovial total nucleated cell count (TNCC) following intra-articular MSC injection.

**Methods:**

Stemness, quantified by a trilineage differentiation (TLD) score; immunomodulation, quantified by mixed lymphocyte reactions (MLRs); and degree of MHCI expression, quantified by mean fluorescent intensity (MFI); were correlated to the synovial TNCC 24 h after naïve and primed injection.

**Results:**

There was a trend of a negative correlation (*p* = 0.21, *r* = − 0.44) between TLD score and TNCC after primed injection in the ALLO group. Within the ALLO group only, there was a significant positive correlation (*p* = 0.05, *r* = 0.77) between MHCI MFI and TNCC after naïve injection and a trend (*p* = 0.16, *r* = 0.49) of a positive association of MHCI MFI to TNCC after primed injection. Within the FBS group only, there was a positive correlation (*p* = 0.04, r = 1) between TNCC and lymphocyte proliferation after both injections.

**Conclusions:**

The trend of a negative correlation of TLD score and TNCC in the ALLO, but not the FBS group, together with the association of MHCI expression and TNCC in the ALLO group, indicates that improved stemness is associated with reduced MSC immunogenicity. When inflammation was incited by xenogen, there was a strong correlation of lymphocyte activation in vitro to adverse response in vivo, confirming that MLRs in vitro reflect MSC immunomodulatory activity in vivo. The relationship of stemness in vitro, suppression of lymphocyte activation in vitro, MHCI expression in vitro, and clinical response in vivo should be further investigated.

## Background

For decades, mesenchymal stem cells (MSCs) have been considered an immune-privileged cell that did not require donor-recipient matching prior to allo-transplantation. This was widely accepted due to the immunologic properties of MSCs in vitro: suppression of stimulated T cells, lack of MHCII expression, relatively low MHCI expression, and production of immunosuppressive cytokines [1]. In other words, MSCs have been understood to have both immunomodulatory properties that reduce inflammation and to lack immunogenic properties that influence allo-recognition. However, recent evidence in the mouse and horse has demonstrated induction of both cellular and humoral immune responses [2–5] and rejection of allo-transplanted MSCs from immune-competent recipients of MHC-mismatched allogeneic MSCs indicating allo-recognition [6].

In a large animal model, we recently demonstrated a local inflammatory response secondary to immune recognition of MSCs after repeated intra-articular injection of allogeneic but not autologous MSCs [7]. In that report, we also demonstrated an inflammatory response to autologous MSCs when MSCs had intracellular xenogen (fetal bovine serum; FBS) contamination. As expected, there was variability in the degree of local inflammation between horses after intra-articular injection within each group. Given the preponderance of data supporting the immunomodulatory function of MSC post-transplant, we suspected the variability of adverse response from each recipient was a result of variability of the immunomodulatory properties of donor MSCs. Specifically, that MSCs from some donors would reduce inflammation to a greater degree than MSCs from other donors. To assess for donor differences in immunomodulatory capacity, we used modified mixed lymphocyte reactions. But to assess for donor differences due to in vitro stemness, we needed an in vitro measure of MSC quality.

Many others have attempted to quantify stem cell quality to predict MSC efficacy and function using in vitro measurements such as motility, growth rate, and various measures of immunomodulatory function [8–11]. In vitro, the MSC is defined by its ability to undergo trilineage differentiation [12], and in vivo, MSCs are thought to remain quiescent in their stem cell niche while maintaining their differentiation potential [13, 14]. Therefore, better maintenance of this inherent MSC characteristic (trilineage differentiation) of MSCs in vitro may reflect better MSC quality. Our objective was to determine if there was a relationship between MSC ability to undergo trilineage differentiation, MSC ability to suppress lymphocyte activation, or amount of MHCI expression in vitro and the in vivo adverse clinical reaction to MSCs from the horses in the aforementioned report.

## Methods

Data and bone marrow-derived MSCs from a previous study that was approved by the university’s Institutional Animal Care and Use Committee (IACUC # 2013-097) were used [7]. For the study reported here, whole blood was collected from the same animals for DNA analysis and haplotype identification (IACUC # 2015-0038). No animals were euthanized for the previous study or the study reported here.

### Study design

Cytologic evaluations of synovial fluid and trilineage differentiation of MSCs from the abovementioned study were used. In that study, horses were assigned to one of three treatment groups: autologous MSCs depleted of FBS (AUTO, *n* = 6), autologous MSCs not depleted of FBS (FBS, *n* = 6), or allogeneic MSCs depleted of FBS (ALLO, *n* = 6). In the allogeneic (ALLO) group, MSCs from one autologous (AUTO) horse were injected in an allogeneic recipient, for a total of six AUTO/ALLO pairs. The metacarpophalangeal joint was injected with approximately 10 × 10^6^ MSCs, respective of the assigned treatment group on day 0 (naïve injection) and day 29 (primed injection). Synovial fluid samples from 24 h after the naïve and primed MSC injection were evaluated.

### Trilineage differentiation scoring

**Fig. 1:**
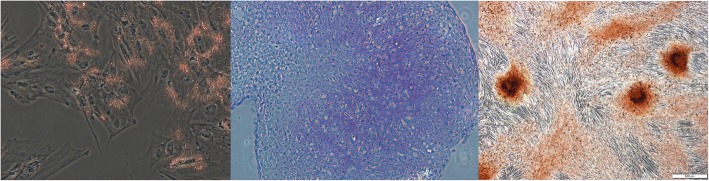
Representative high scoring images for adipogenesis, chondrogenesis, and osteogenesis. All images were taken at × 10 magnification

A composite trilineage differentiation (TLD) score, a measure of stemness, was produced for all 12 donors (AUTO, *n* = 6; FBS, *n* = 6) from the average scores by a blinded individual for all lineages. Chondrogenesis was evaluated using the Bern Score based on the uniformity of stain uptake, cell density, and cell morphology with each category having a maximum of 3, making 9 the total maximum chondrogenesis score (Table 1) [15]. The degree of osteogenesis was scored from 0 to 4 based on the percentage of cells with alizarin red^1^ uptake in six randomized fields at a × 10 objective on three replicate plates. Adipogenesis was also scored from 0 to 4 based on the percentage of cells with oil red O^2^ uptake; six randomized areas were scored in three replicate wells with the mean value being the adipogenesis score (Fig. 1). Each lineage was adjusted to a maximum score of 4 to weight each lineage equally, resulting in a total maximum total TLD score of 12. Mesenchymal stem cells were considered bipotent if TLD scores from one lineage were equal to or less than one.

**Table 1:** Trilineage differentiation scoring rubric for all lineages. Chondrogenesis rubric based on the Bern Score [15]

Lineage	Score
Chondrogenesis	
Uniformity and darkness of stain	
No stain	0
Weak staining of poorly formed matrix	1
Moderately even staining	2
Even dark stain	3
Distance between cells/amount of matrix accumulated	
High cell densities with no matrix between	0
High cell densities with a little matrix in between	1
Moderate cell density with matrix	2
Low cell density with a moderate distance between	3
Cell morphologies represented	
Condensed/necrotic/pycnotic bodies	0
Spindle/fibrous	1
Mixed spindle/fibrous with rounded chondrogenic morphology	2
Majority rounded/chondrogenic	3
Adipogenesis	
0% of cells with stain uptake	0
1–25% of cells with stain uptake	1
26–50% of cells with stain uptake	2
51–75% of cells with stain uptake	3
76–100% of cells with stain uptake	4
Osteogenesis	
0% of cells with stain uptake	0
1–25% of cells with stain uptake	1
26–50% of cells with stain uptake	2
51–75% of cells with stain uptake	3
76–100% of cells with stain uptake	4

### MHC haplotype analysis

Haplotype analysis was performed on the recipients in the ALLO group, as well as donors from the AUTO group. Lymphocytes were isolated from the peripheral blood using a Ficoll^3^ gradient as previously described and DNA extracted using a commercially available kit^4^ [4]. Ten microsatellite loci, components of MHC I, II, or III, were analyzed to determine the haplotype of the individual. Briefly, this was done using the previously described method of amplifying genomic DNA using multiplex PCR with known primers of different loci and analyzing the size of the resulting products with commercially available software^5^ [16]. Haplotypes not previously established were reported as unknown, or as novel if two or more other individuals with the same haplotype had been identified.

### Population doubling time

Cell counts and time in culture during expansion of MSCs for the previous study was used to compute population doubling time (PDT) from passages 2–3 as a previously described measure of growth rate [17]. The following formula was used: PDT = Time in culture × log 2/(log_*f*_ − log_*i*_) where *f* is final cell count and *i* is the initial number of cell.

### MHCI expression

Cryopreserved cells were thawed and CZ3.2 antibody^6^ was added undiluted to one million cells and incubated for 45 min at room temperature. Cells were then washed, and secondary antibody^7^ was added at a 1:100 dilution and incubated again for 45 min, then washed twice and resuspended in DPBS before flow cytometry was performed. Unstained MSCs and unstained MSCs with only secondary antibody added were used as controls.

### Mixed lymphocyte reactions

Cryopreserved cells were thawed and plated at a density of 50,000 cells per well 24 h prior to inactivation with mitomycin C^8^ as previously described [18]. Responder and stimulator lymphocytes were isolated from two unrelated donors, respectively, using a Ficoll^3^ gradient with the addition of carbonyl iron^9^ [19]. Stimulator lymphocytes were also inactivated by incubation with 50 μg/ml mitomycin C for 30 min and then added at a density of 1 × 10^6^ stimulator lymphocytes per well. Responder lymphocytes were stained with a commercially available nuclear stain,^10^ and 2 × 10^6^ responder lymphocytes were added to each well. Cultures were maintained for 5 days in 1640 RPMI lymphocyte culture media. After 5 days, lymphocytes were collected and stained with anti-equine CD3+ antibody^11^ at a 1:200 dilution. Flow cytometry was then performed on CD3+ T lymphocytes to assess proliferation with the use of the commercially available software.^12^ Stained, unstimulated responder lymphocytes were used as a negative proliferation control, and changes in mean fluoresce intensity were evaluated as a percent change from the negative control as previously described [20].

### Statistical analysis

Spearman correlation coefficients were used to assess correlation in the AUTO, FBS, and ALLO groups of the TLD score to day 1 and day 30 TNCC. As a follow-up to immunogenicity versus immunomodulatory capacity of MSCs, the MFI of MHCI expression and the percent change of MFI of responder lymphocytes were compared to TNCC also using Spearman correlation coefficients. Stemness scores were also tested for a correlation using Spearman correlation coefficients to MFI of MHCI expression and PDT. Differences between the groups in TLD score, MFI of MHCI expression, and the percent change of MFI of responder lymphocytes were analyzed using a Wilcoxon rank sum test. A commercially available statistical software^13^ was utilized and differences considered significant when *p* ≤ 0.05.

## Results

### Trilineage differentiation scores

**Fig. 2:**
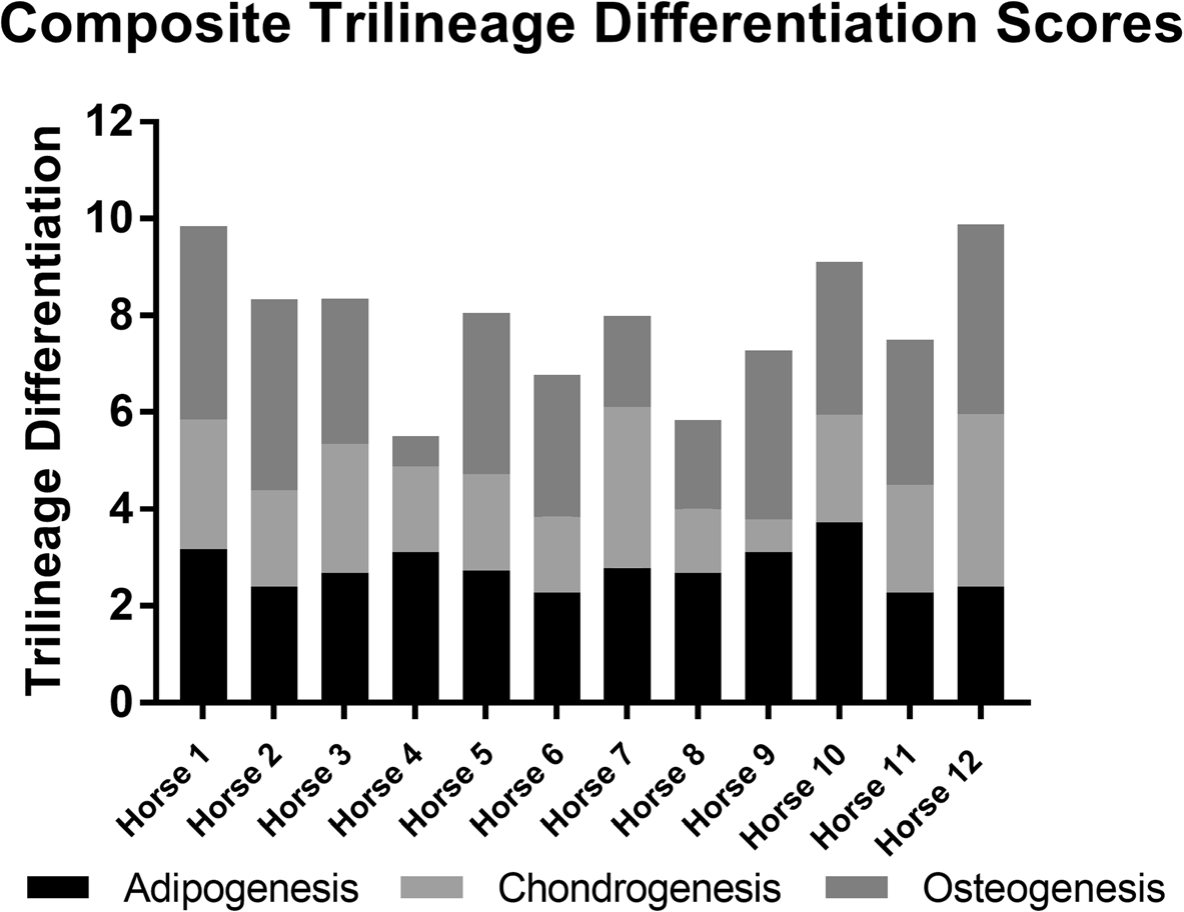
Stacked trilineage differentiation (TLD) scores showing MSC differentiation from each donor in the AUTO and FBS groups. The black portion of the bar represents adipogenesis, light gray represents chondrogenesis, and dark gray represents osteogenesis. Each cell lineage was weighted equally with a maximum score of 4. Horses 1–6 (AUTO)received autologous cell depleted of FBS and were donors for horses in the ALLO group. Horses 7–12 (FBS) received autologous cells contaminated with intracellular xenogen. There were no significant differences between the groups

**Fig. 3:**
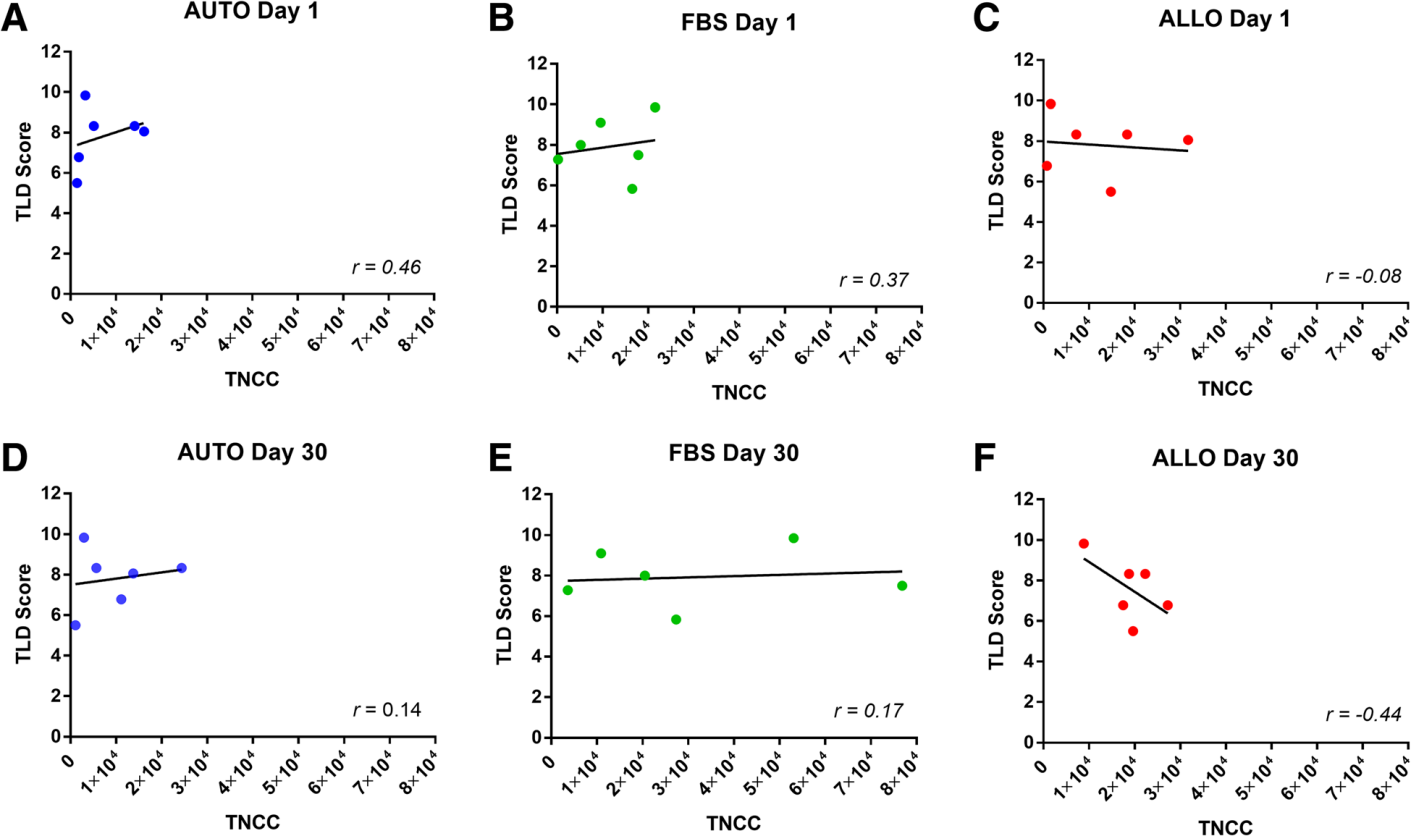
Scatter plots of trilineage differentiation (TLD) scores compared to TNCC in each group after the naive inection, **a**–**c** and after the primed injection **d-f**.  **a-c** The inflammatory response (measured by TNCC) after the first injection had no significant correlations to TLD score (AUTO, *p* = 0.18; FBS, *p* = 0.37; ALLO, *p* = 0.43). **d**–**f** The inflammatory response after the second injection had no significant correlation to TLD score in the AUTO or FBS group (AUTO, *p* = 0.37; FBS, *p* = 0.40). A trend of a negative correlation was noted in the ALLO group after the second injection (*p* = 0.21, *r* = − 0.44)

TLD scores ranged from 5.5 to 9.7 out of 12 with a median score of 8. There was no significant difference in the scores between the AUTO and FBS groups (*p* = 0.5) (Fig. 2). After the naïve intra-articular injection (day 1), there were no significant correlations between TLD and TNCC (AUTO, *p* = 0.18; FBS, *p* = 0.25; ALLO, *p* = 0.43) (Fig. 3a–c). After the primed intra-articular injection (day 30), there were no significant correlations between TLD and TNCC in the AUTO or FBS group (AUTO, *p* = 0.37; FBS, *p* = 0.40), but there was a trend (*p* = 0.21) of a negative correlation (*r* = − 0.44 in the ALLO group (Fig. 3d–f). Horse 4 (AUTO/ALLO group) and horse 9 (FBS group) were bipotent, receiving a TLD score of 1 for one lineage. Removal of horses 4 and 9 from the analysis did not change the direction or statistical significance of the result but did result in a more negative correlation in the ALLO group after second intra-articular injection (ALLO, *p* = 0.23, *r* = − 0.53; AUTO, *p* = .21, *r* = − 0.46; FBS, *p* = 0.39, *r* = − 0.2).

### MHC haplotype analysis

All MSC donors were MHC mismatches to their recipient (Table 2). Three horses in the ALLO group received cells from an individual that was a complete mismatch (both alleles were different), and three horses received cells from an individual that was a partial mismatch (one allele was similar, one was different). There was no significant difference in TNCC post-injection by Wilcoxon rank sum between the horses that were partial mismatches versus total mismatches.

**Table 2:** Mean fluorescence intensity and percentage of cells positive for MHCI. Horses 1–6 comprised the AUTO group and were donors for single recipients in the ALLO group; horses 7–12 were in the FBS group

	Mean fluorescence intensity	Percentage of positive cells
Horse 1	23.5	91.1
Horse 2	88.2	99.9
Horse 3	129	100
Horse 4	426	100
Horse 5	188	99.8
Horse 6	68.5	100
Horse 7	43.7	97.9
Horse 8	79.9	99.7
Horse 9	101	99.9
Horse 10	85.1	99.9
Horse 11	211	100
Horse 12	93.1	99.9

### Population doubling time

Population doubling time was not different between the groups (AUTO, median 3.1 days; FBS, median 1.7 days). There was no correlation between PDT and TLD score in either group.

### MHCI expression

**Fig. 4:**
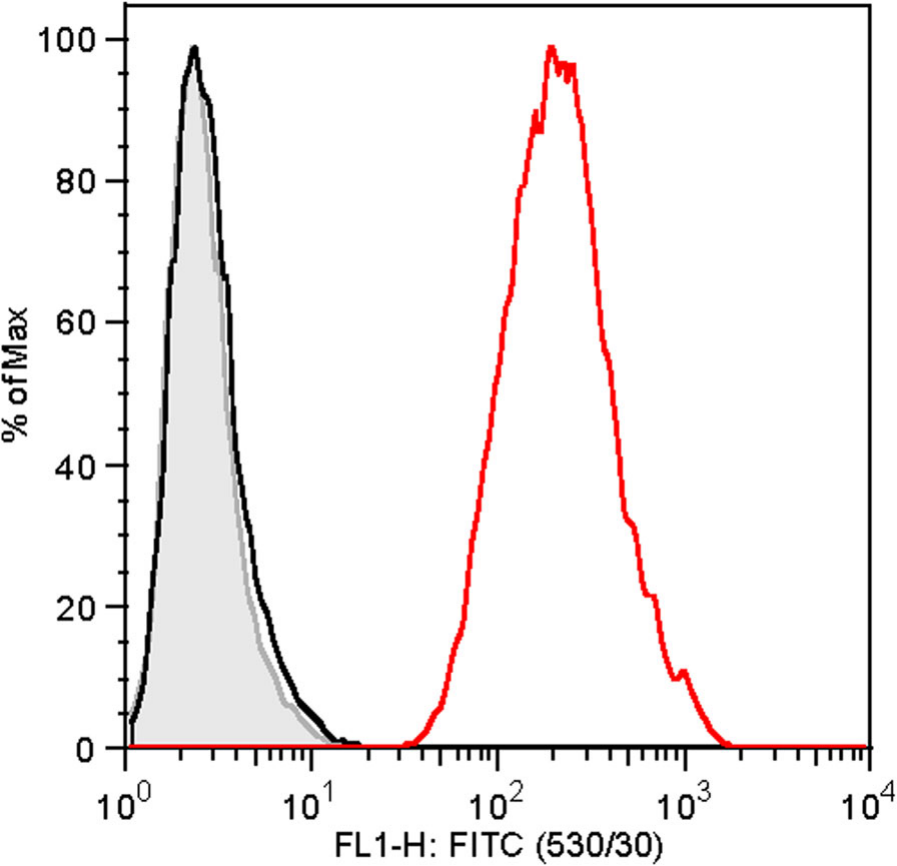
Representative histogram of MHCI analysis. The gray area represents the unstained control, black the unstained control with secondary antibody only added, and red the sample cell stained with CZ3.2 and anti-MHCI antibody, as well as secondary FITC-conjugated antibody

MSCs from all donors in the AUTO and FBS groups were positive for MHCI expression (percentage of cells expressing MHCI median, 99.9%; range, 91.1–100%) (Fig. 4). Mean fluorescent intensity (MFI) ranged widely between individuals (median, 88.2; range, 23.5–426) (Table 3). There was no correlation between MHCI MFI and TLD (*r* = − 0.26). There was no correlation between MHCI MFI and TNCC in the AUTO or FBS group at either time point. In the ALLO group, there was a significant positive correlation (*p* = 0.05, *r* = 0.77) between MHCI MFI and TNCC at day 1 as well as a positive trend at day 30 (*p* = 0.16, *r* = 0.49).

**Table 3:** Haplotype analysis of horses 1–6 and 13–18. Horses 1–6 comprised the AUTO group and were donors for the ALLO group; horses 13–18 were the ALLO group and recipients of MSCs from horses in the AUTO group. There were no complete matches between donor and recipient

MHC class	I	I	III	III	II	II	II	II	II	II	
Microsatellite loci	UMNJH-38	COR110	ABGe9019	UMNe65	ABGe9030	EQMHC1	COR112	COR113	UM011	COR114	
											Haplotype
AUTO group (donors)											
Horse 1	165	219	316	257	206	192	256	270	174	234	Nov_07
	156	211	318	263	215	194	256	270	178	245	Nov_05
Horse 2	156	221	299	257	212	190	254	260	172	243	A5a
	156	211	299	257	205	192	260	268	172	251	Nov_03
Horse 3	165	211	314	259	206	190	264	268	169	259	Nov_06
	156	221	314	259	205	194	256	270	172	249	QHnov04
Horse 4	**	221	312	261	207	190	237	264	180	243	A10a
	**	211	314	259	206	190	264	268	169	259	Unknown
Horse 5	156	221	314	259	205	194	256	270	172	249	QHnov04
	156	211	318	257	212	190	262	270	184	245	A19
Horse 6	156	209	297	269	205	194	258	260	169	243	Nov_02
	156	221	314	259	205	194	256	270	172	249	QHnov04
ALLO group (recipients)											
Horse 13	156	221	299	257	212	190	254	260	172	243	A5a
	156	221	314	259	205	194	256	270	172	249	QHnov04
Horse 14	156	221	299	257	212	190	254	260	172	243	A5a
	156	211	301	263	211	184	252	260	169	243	Nov_01
Horse 15	156	221	299	257	212	190	256	260	172	243	A5A
	156	211	318	257	215	194	256	270	178	245	Nov_05
Horse 16	156	209	297	269	205	194	258	260	169	243	Nov_02
	156	211	301	263	211	184	252	260	169	243	Nov_01
Horse 17	156	211	299	257	207	190	237	266	179	241	A1
	156	221	314	259	205	194	256	270	172	249	QHnov04
Horse 18	156	221	299	257	212	190	254	260	172	243	A5a
	156	221	299	257	212	190	254	260	172	243	A5a

Asterisks indicate when microsatellite loci could not be determined

### Mixed lymphocyte reactions

**Fig. 5:**
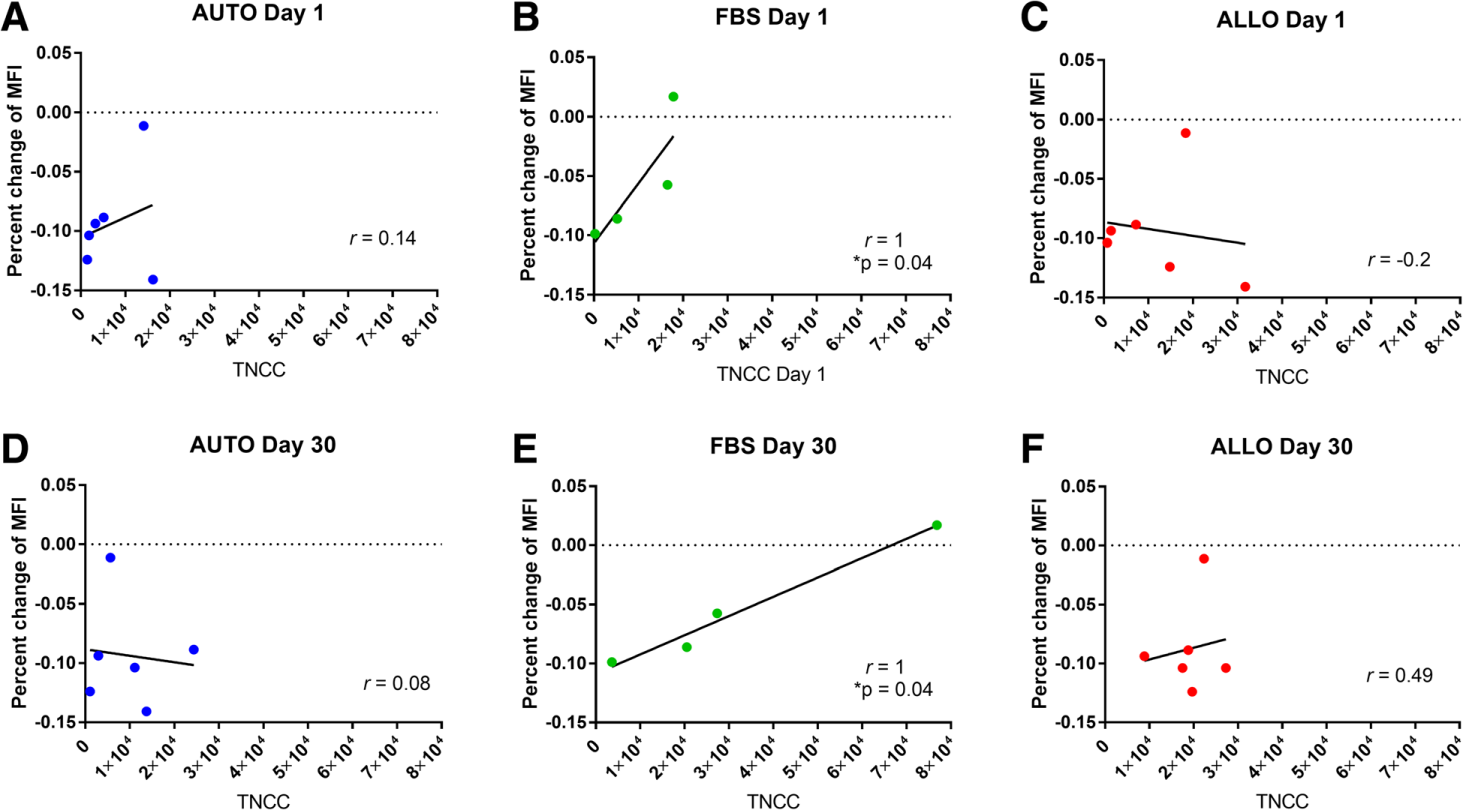
Scatter plots from mixed lymphocyte reactions measuring MSC immunomodulatory capacity shown as percent change in mean fluorescence intensity (MFI) compared to TNCC in each group **a**-**c** after the naive injection and **d-f** after the primed injection. Unstimulated responder lymphocytes were used to calculate percent change. There were no significant correlations in the AUTO or ALLO group at either time points (day 1: AUTO *p* = 0.40, ALLO *p* = 0.36; day 30: AUTO *p* = 0.46, ALLO *p* = 0.49). A significant correlation was noted in the FBS group after both injections (day 1, *p* = 0.04; day 30, *p* = 0.04)

MSCs from two horses in the FBS group were unavailable to complete mixed lymphocyte reactions. There was no significant correlation between TLD score and percent change in mean fluorescence intensity (MFI) of responder lymphocytes in either group (AUTO and FBS) or overall. There was no significant correlation between TNCC and percent change in MFI in the AUTO or ALLO group after first or second injection (Fig. 5). There was a significant correlation between TNCC and percent change in mean fluorescence intensity (MFI) in the FBS group after both first and second injection (day 1, *p* = 0.04, *r* = 1; day 30, *p* = 0.04, *r* = 1) (Fig. 5)

## Discussion

We previously demonstrated that repeated intra-articular injection of intracellular xenogen-contaminated autologous MSCs and allogeneic MSCs without intracellular xenogen resulted in an adverse clinical response after the second intra-articular injection in horses [7]. In that report, there was a significant intra-articular inflammation following the second injection because of immune recognition of foreign antigens: either allogen or xenogen.

Our objective for the study reported here was to use data and MSCs from that study to ascertain the relationship between stemness, immunomodulatory properties and recipient response. We expected that we would find a significant negative correlation of TLD score to TNCC, our measure of intra-articular inflammation, in both inflamed groups because of enhanced immunomodulatory properties of more plastic MSCs. However, we found a trend of a correlation only in the allogeneic group. The trend of a negative correlation in the ALLO group suggested reduced MSC immunogenicity with improved MSC stemness. To further investigate this trend, we evaluated the degree of MHCI expression on all MSCs (AUTO and FBS). We found a negative correlation of MHCI expression to TNCC in the ALLO group and no relationship with TLD scores in any group. It has been suggested by others that MSCs with a greater degree of stemness express less MHCI [21], and our data supports that MHCI expression on allogeneic MSCs impacts adverse recipient response [22].

The degree of MHCI expression is an important MSC characteristic [23]. Adult-derived MSCs are known to have reduced MHCI expression compared to other somatic cells, and this MSC characteristic has contributed to the immune-privileged label that MSCs are often given [24]. Although low, the expression of MHCI was ubiquitously positive from MSCs in both groups. This is not surprising, as some MHCI expression is required because lack of MHCI will lead to recognition and destruction of any cell within the host by natural killer cells [25]. The importance of the degree of MHCI expression has been recognized and investigated. Berglund et al. utilized TGF-β2 to reduce MHCI expression in an effort to minimize allo-recognition of MSCs [26]. Our work testing the recipient response to MHC mismatched MSC injections supports this notion that a reduction in MHCI expression by MSCs may reduce allo-recognition, and degree of MHCI expression by MSCs may be an important measure of MSC quality. While we understand that MHC-mismatched allogeneic MSCs elicit an inflammatory reaction, the immune distance between haplotypes is unknown at this time, making the degree of mismatch between different haplotypes difficult to interpret (Table 3).

Interestingly, when evaluating mixed lymphocyte reactions (MLRs), we found no relationship of stemness to immunomodulatory function. However, we did find a significant correlation between in vitro MLRs and in vivo TNCC in the FBS group after both the first and second injections. This confirms the validity of MLRs in predicting MSC function in the inflamed environment in vivo; when there is a significant inflammation, which in our case was induced by introduction of intracellular xenogen within MSCs, the immunomodulatory capacity of the MSC in vitro is related to its immunomodulatory capacity in vivo.

We chose TLD to quantify MSC quality as it is a basic defining characteristic of an MSC and is easily quantified. Others have used trilineage differentiation ability as a measure of MSC quality and found that it may predict efficacy [10]. We expected that our TLD scores might be a good measure of MSC quality to predict their immune-modulating properties in vivo. Instead, we found that trilineage differentiation ability was related to MSC immunogenicity but not immunomodulatory properties.

One limitation of using a TLD score is that bipotent MSCs receiving a high score for two of the three tissue types could receive a higher score than tripotent MSCs with moderate scores for all three lineages. Horse 4 and horse 9 received a score of less than or equal to 1 in a single lineage, suggesting a bipotent instead of tripotent differentiation capability. Because we sought to evaluate stemness, thus the ability to differentiate equally into all three lineages, we removed the associated bipotent scores from the analyses, which did not change the results. In future studies using a TLD score, it will be important to consider the effect of a strongly bipotent MSC. Another limitation of our TLD score was the small range covered by the TLD scores and the ordinal nature of the data. A validated assay of trilineage differentiation ability that produced continuous data should be developed.

## Conclusions

It is becoming clear that MSCs are not as immune privileged as was once thought. An improved understanding of the factors that contribute to MSC immunogenicity is needed and may lead to ex vivo techniques to minimize immunogenicity and enhance the therapeutic efficacy of allogeneic MSCs. Overall, we demonstrated that in vitro stemness might be related to allo-MSC immunogenicity but not immunomodulatory function, in vivo. We confirmed that MLRs in vitro reflect MSC immunomodulatory function in vivo, when the inflammatory stimulus is intracellular xenogen. The relationship of in vitro stemness, in vitro suppression of lymphocyte activation, MHCI expression, and clinical reaction in vivo should be further investigated.

## Abbreviations

ALLO: Allogeneic
AUTO: Autologous
FBS: Fetal bovine serum
MSCs: Mesenchymal stem cells
PDT: Population doubling time
TLD: Trilineage differentiation
TNCC: Total nucleated cell count

## Footnotes

1 Sigma Aldrich, St. Louis, MO

2 Sigma Aldrich, St. Louis, MO

3 Ficoll Paque, GE Healthcare, Aurora, OH

4 DNeasey, Qiagen, Germantown, MD

5 Biosystems Peakscanner software v1.0, Applied Biosystems Foster City, California, USA

6 Antczak Laboratory, Baker Institute, Ithaca, NY

7 Fluorescein-AffiniPure Goat-Anti Mouse IgG, Jackson ImmunoResearch, West Grove, PA

8 Sigma Aldrich, St. Louis, MO

9 Sigma Aldrich, St. Louis, MO

10 CellTrace™ Violet, Thermo Fisher Scientific, Waltham, MA

11 Clone UC F6G, University of California, Davis, CA

12 FlowJo, FlowJo LLC, Ashland, OR

13 Prism, GraphPad, La Jolla, CA
